# Timing of tracheostomy as a determinant of weaning success in critically ill patients: a retrospective study

**DOI:** 10.1186/cc3018

**Published:** 2004-12-23

**Authors:** Chia-Lin Hsu, Kuan-Yu Chen, Chia-Hsuin Chang, Jih-Shuin Jerng, Chong-Jen Yu, Pan-Chyr Yang

**Affiliations:** 1 Division of Pulmonary Medicine, Department of Internal Medicine, National Taiwan University Hospital, Taipei, Taiwan; 2 Division of General Medicine, Department of Internal Medicine, National Taiwan University Hospital, Taipei, Taiwan; 3 Assistant Professor, Division of Pulmonary Medicine, Department of Internal Medicine, National Taiwan University Hospital, Taipei, Taiwan; 4 Professor, Division of Pulmonary Medicine, Department of Internal Medicine, National Taiwan University Hospital, Taipei, Taiwan

**Keywords:** critical illness, mechanical ventilation, tracheostomy, weaning

## Abstract

**Introduction:**

Tracheostomy is frequently performed in critically ill patients for prolonged intubation. However, the optimal timing of tracheostomy, and its impact on weaning from mechanical ventilation and outcomes in critically ill patients who require mechanical ventilation remain controversial.

**Methods:**

The medical records of patients who underwent tracheostomy in the medical intensive care unit (ICU) of a tertiary medical centre from July 1998 to June 2001 were reviewed. Clinical characteristics, length of stay in the ICU, rates of post-tracheostomy pneumonia, weaning from mechanical ventilation and mortality rates were analyzed.

**Results:**

A total of 163 patients (93 men and 70 women) were included; their mean age was 70 years. Patients were classified into two groups: successful weaning (*n *= 78) and failure to wean (*n *= 85). Shorter intubation periods (*P *= 0.02), length of ICU stay (*P *= 0.001) and post-tracheostomy ICU stay (*P *= 0.005) were noted in patients in the successful weaning group. Patients who underwent tracheostomy more than 3 weeks after intubation had higher ICU mortality rates and rates of weaning failure. The length of intubation correlated with the length of ICU stay in the successful weaning group (r = 0.70; *P *< 0.001). Multivariate analysis revealed that tracheostomy after 3 weeks of intubation, poor oxygenation before tracheostomy (arterial oxygen tension/fractional inspired oxygen ratio <250) and occurrence of nosocomial pneumonia after tracheostomy were independent predictors of weaning failure.

**Conclusion:**

The study suggests that tracheostomy after 21 days of intubation is associated with a higher rate of failure to wean from mechanical ventilation, longer ICU stay and higher ICU mortality.

## Introduction

Tracheostomy is among the most frequently performed procedures in critically ill patients, being done in about 24% of patients in medical intensive care units (ICUs) [1]. The most common indication for tracheostomy in the ICU is need for prolonged mechanical ventilation [2,3]. Tracheostomy has several advantages over endotracheal intubation, including lower airway resistance, smaller dead space, less movement of the tube within the trachea, greater patient comfort and more efficient suction [4,5]. Although recent studies have suggested that tracheostomy can be a safe procedure in the ICU [6,7], tracheostomy has also been found to lead to serious complications, including tracheal stenosis, increased bacterial colonization and haemorrhage [8,9]. Many critically ill patients' families have been hesitant in authorizing tracheostomy because of cosmetic issues and speech problems.

Because there are no definitive guidelines available, the timing of tracheostomy depends on clinical conditions, physician judgement and communication with families. The judgement of the attending physician can be influenced by the patients' likelihood of extubation, life expectancy and other clinical conditions, including haemodynamic status, oxygenation, consciousness level and ability to protect the airway. There is little consensus on the timing of tracheostomy. In the 1989 American College of Chest Physicians (ACCP) Consensus Conference on Artificial Airways in Patients Receiving Mechanical Ventilation [10], it was concluded that the appropriate duration of translaryngeal intubation could not be defined. It was suggested that if the anticipated need for mechanical ventilation is longer than 21 days then tracheostomy is preferable. For mechanical ventilation that is anticipated to last between 10 and 21 days, the decision was left to the physician, and daily assessment was recommended. Recent ACCP guidelines [11] suggest that tracheostomy should be considered after an initial period of stabilization on the ventilator, when it becomes apparent that the patient will require prolonged ventilator assistance.

Maziak and coworkers [12] reviewed five reports on the timing of tracheostomy and concluded that there was insufficient evidence to conclude that the timing of tracheostomy alters the duration of mechanical ventilation. However, there is still a lack of data on the relationship between the timing of tracheostomy and weaning from mechanical ventilation for patients in the medical ICU. Therefore, we investigated the timing of tracheostomy and other factors that might influence weaning from mechanical ventilation and outcomes of patients admitted to the medical ICU.

## Methods

### Patients

Over a period of 36 months (from July 1998 to June 2001), all adult patients admitted to the medical ICU of National Taiwan University Hospital – a 1500-bed tertiary medical centre that accommodates tracheostomy within the ICU – were considered for inclusion in the study. Patients were excluded if the tracheostomy was performed in an emergency setting because of difficulties with the airway or other causes. Tracheostomy was performed using standard surgical techniques at bedside in the ICU, and no patients underwent percutaneous tracheostomy. The timing of tracheostomy depended on the attending physician's decision. Indications to initiate an attempt to wean a patient from mechanical ventilation included stable haemodynamic status, improved oxygenation (arterial oxygen tension [PaO_2_]/fractional inspired oxygen [FiO_2_] ratio >150), controlled infection and lack of need for further intervention. The weaning process was begun with synchronized intermittent mandatory ventilation with pressure support. Then, patients underwent continuous positive airway pressure with pressure support, or intermittent T-piece for a spontaneous breathing trial when clinical conditions improved. Successful weaning was defined as weaning from mechanical ventilation for more than 72 hours. Patients were transferred to long-term care settings once tracheostomy and the weaning process were completed if there was no other active clinical disease.

### Data collection

The indications for intubation were defined as any major problem(s) that necessitated intubation. The underlying disease of the patients, including diabetes mellitus, hypertension, congestive heart failure, chronic renal insufficiency, chronic obstructive pulmonary disease and malignant disease with lung metastasis, were ascertained through chart reviews. Medical records were analyzed for age, sex, underlying disease and cause of intubation, Acute Physiology and Chronic Health Evaluation (APACHE) II score [13], duration of mechanical ventilation, complications of tracheostomy, pneumonia after tracheostomy, length of ICU stay, and mortality in the ICU and hospital. APACHE II scores were calculated using clinical data, which were available from the first 24 hours of intensive care. Clinical data within 72 hours before tracheostomy, including PaO_2_/FiO_2 _ratio, peripheral white blood cell (WBC) counts, haemoglobin, creatinine and albumin, were also recorded and analyzed. Old age was defined as age above 65 years. Anaemia was defined as haemoglobin below 10 g/dl, and leucocytosis was defined as a WBC count above 11,000/ μl before tracheostomy. Renal insufficiency was defined as creatinine above 1.5 mg/dl, and poor oxygenation as PaO_2_/FiO_2 _ratio below 250.

Complications of tracheostomy, including bleeding, air leakage, pneumothorax, subcutaneous emphysema, cardiopulmonary arrest, dislodgement of the tube, obstruction, tracheal stenosis, granuloma, tracheo-oesophageal fistula and tracheomalacia, were recorded. Complications that occurred within 7 days after tracheostomy were defined as early complications; those occurring later were considered late complications. Severity of bleeding after tracheostomy was classified as follows: minor if there was only minimal blood clot over the wound or if new onset bloody sputum was noted on the next day of the tracheostomy; moderate if bleeding needed external compression and component therapy or surgical management; and massive if the bleeding resulted in obvious haemodynamic change. The clinical definition of post-tracheostomy pneumonia used was as follows [14]: new and persistent radiographic opacity found after the tracheostomy had been removed and within 48 hours into the weaning period; positive sputum culture; and three of body temperature above 38°C, WBC count above 15,000/μl, increased airway secretions, or worsening gas exchange.

### Statistical analysis

Values are expressed as mean ± standard deviation (continuous variables) or as a percentage of the group from which they were derived (categorical variables). Only variables with complete data were analyzed in the study. Differences in the groups, including sex, underlying diseases and associated medical conditions, indications for intubation, occurrence of post-tracheostomy pneumonia, successful weaning and mortality, were analyzed using χ^2 ^test. Other variables, including age, sex, APACHE II score, the length of ICU stay, PaO_2_/FiO_2 _ratio, peripheral WBC count, haemoglobin, albumin and weaning period, were analyzed by an independent t-test. The correlations between the intubation period and the length of ICU stay were analyzed using a Pearson bivariate correlation test. The correlations between successful weaning and potentially influential factors, including old age, sex, presence of comorbidities, indications for intubation, leucocytosis, anaemia, thrombocytopenia, renal insufficiency, poor oxygenation, post-tracheostomy pneumonia and timing of tracheostomy, were analyzed using the Kaplan–Meier method with a log rank test. Censoring was performed for those patients who died during mechanical ventilation. A Cox regression model was applied for multivariate analysis with variables that were significantly associated with successful weaning in the univariate analysis. *P *< 0.05 was considered statistically significant.

## Results

### Clinical characteristics

From July 1998 through June 2001, a total of 167 patients who underwent tracheostomy in the medical ICU were included in the study. Four patients were excluded because of emergent tracheostomy due to difficult airway (*n *= 3) or laryngeal oedema (*n *= 1). Thus, 163 patients were included (93 male and 70 female; mean age 70 years, range 19–104 years; Table 1). The indications for intubation in the 163 patients were classified into four categories: pulmonary (*n *= 107), infectious (*n *= 18), neurological (*n *= 28) and circulatory (*n *= 10) disease. The most common cause of intubation was pneumonia with respiratory failure (*n *= 81 [73%]). The mean APACHE II score within the first 24 hours after ICU admission was 20.0 ± 7.2. The mean duration of intubation was 18.5 ± 10.9 days (range 1–62 days).

### Complications

The most common early complication of tracheostomy was bleeding (moderate bleeding in 11 [6.7%] and minor bleeding in 46 [28.2%]), followed by subcutaneous emphysema (3 [1.8%]; in two this occurred together with bleeding and in one it occurred together with air leakage) and obstruction (3 [1.8%]). The most common late complication was bleeding (4 [2.5%]), followed by air leakage (3 [1.8%]) and tracheal stenosis (2 [1.2%]). The incidence of complications did not differ significantly between the successful weaning and failure-to-wean groups (early complications: 38.5% versus 37.6%, *P *= 1.0; late complications: 6.4% versus 9.4%, *P *= 0.6). No patient died during the procedure operation or because of complications of tracheostomy.

### Timing of tracheostomy and outcomes

The patients were divided in two groups according to weaning outcome. Seventy-eight patients were successfully weaned from mechanical ventilation, and 85 patients failed to wean. The clinical characteristics, including sex, age, APACHE II score and previous comorbid conditions, were similar between the groups (Table 1). The most frequent reason for intubation was pulmonary disease (107 [65.6%]), followed by neurological disease (28 [17.2%]). The indications for intubation in the two groups were also similar, except that more neurological disease was noted in the successful weaning group (Table 2). Hypoalbuminaemia, anaemia, leucocytosis and impaired gas exchange were noted before tracheostomy. Pre-tracheostomy albumin, creatinine and haemoglobin levels were similar between groups, but the failure-to-wean group was noted to have higher WBC counts (*P *= 0.05), lower platelet counts (*P *= 0.005) and poor PaO_2_/FiO_2 _ratio (*P *= 0.003; Table 3). After tracheostomy, 109 patients (66.9%) developed nosocomial pneumonia. The average number of post-tracheostomy ventilator days was 27.3. Higher rates of post-tracheostomy pneumonia (*P *= 0.05) and longer post-tracheostomy mechanical ventilation periods (*P *= 0.001) were noted in the failure-to-wean group (Table 4). Shorter intubation periods (*P *= 0.02), length of ICU stay (*P *= 0.001) and post-tracheostomy ICU stay (*P *= 0.005) were noted in the successful weaning group (Table 4). The overall ICU mortality was around 19%.

**Figure 1:**
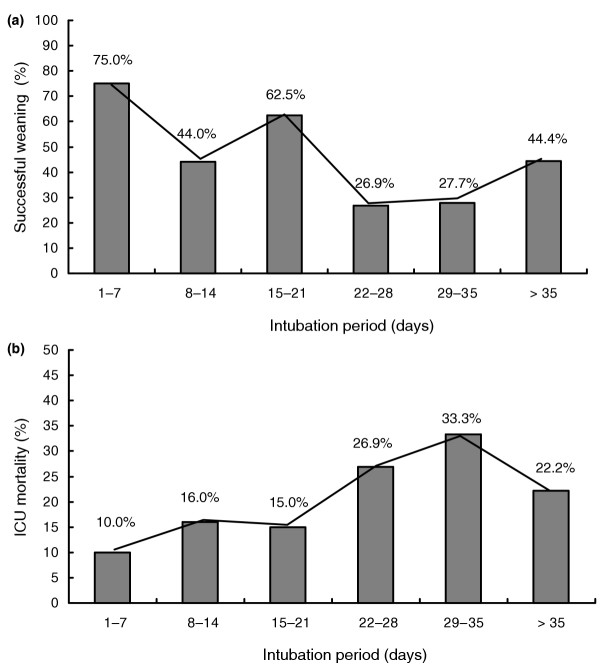
**The relationship of weaning rates, ICU mortality and durations of intubation. (a) **Rate of successful weaning in patients who underwent tracheostomy after different durations of intubation. The rate of successful weaning declined when patients underwent tracheostomy after 21 days of intubation. **(b) **Intensive care unit (ICU) mortality rates in patients who underwent tracheostomy after different durations of intubation. The ICU mortality rates increased when the patients underwent tracheostomy after 21 days of intubation.

**Figure 2:**
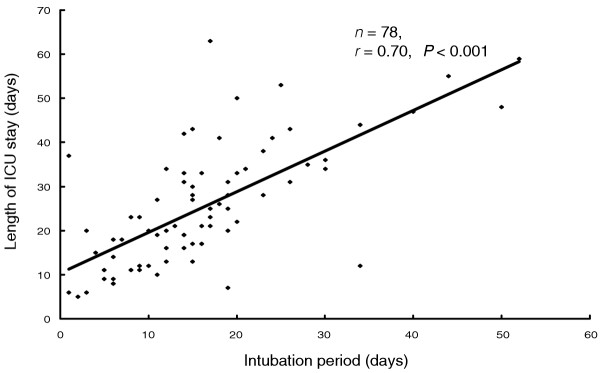
Correlation of intubation period and the length of intensive care unit (ICU) stay in patients who weaned successfully.

ICU mortality is summarized in Fig. 1. Regarding the relationship of timing of tracheostomy to successful weaning, an intubation period in excess of 21 days was associated with decreased rate of successful weaning (31.5% versus 56%, *P *= 0.004) and increased ICU mortality (27.8% versus 14.7%, *P *= 0.057). The intubation period exhibited a correlation with length of ICU stay in the successful weaning group (r = 0.70, *P *< 0.001; Fig. 2). We used day 21 as a cut-off point to define early and late trachostomy, in accordance with the clinical observations summarized in Fig. 1. Early tracheostomy was defined as tracheostomy performed within 21 days after intubation (*n *= 110); late tracheostomy was defined as tracheostomy performed later than this (*n *= 53). The early tracheostomy patient group had a higher rate of successful weaning (56.4% versus 30.2%, *P *= 0.002) and lower ICU mortality (14.5% versus 28.3%, *P *= 0.05), but there were no differences between early and late tracheostomy groups in terms of hospital mortality (44.5% versus 54.7%, *P *= 0.25) or occurrence of nosocomial pneumonia during the weaning period (43.6% versus 60.4%, *P *= 0.06). The patients who underwent early tracheostomy also had shorter post-tracheostomy ICU stays (10.8 versus 14.2 days, *P *= 0.04) and weaning periods (19.0 versus 44.3 days, *P *< 0.001).

**Figure 3:**
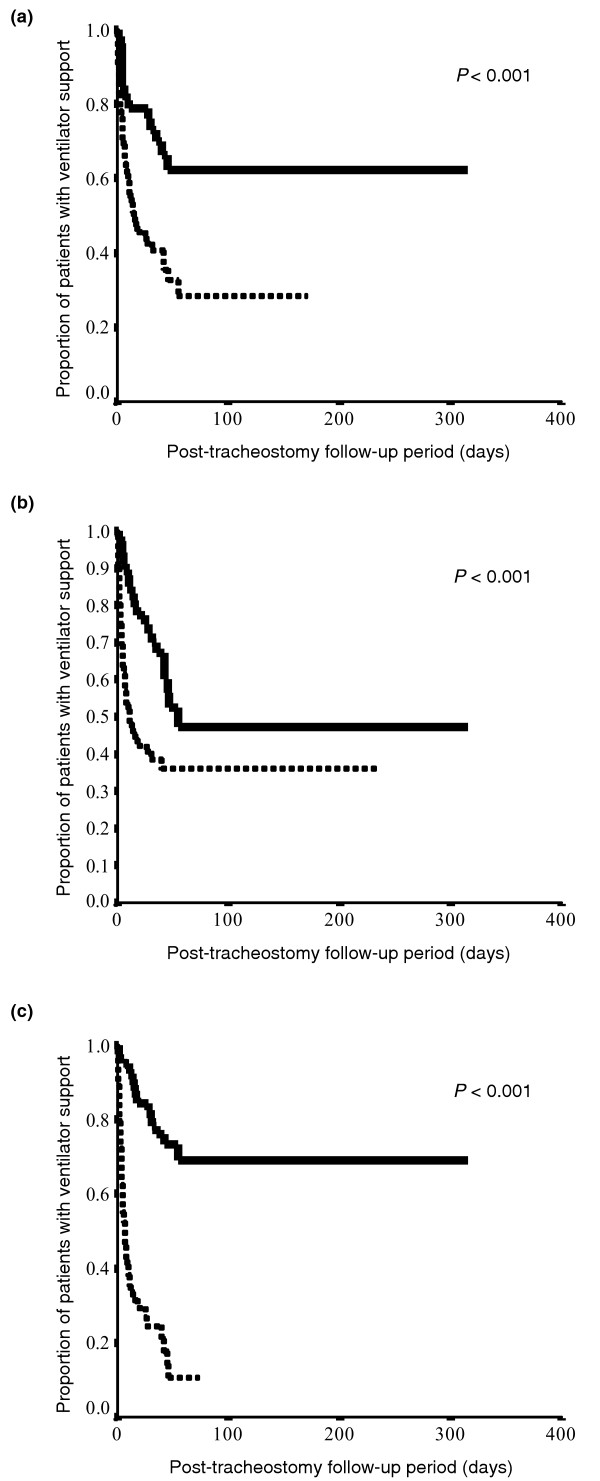
**Survival curves of independent predictors of weaning failure. (a) **Difference in rates of successful weaning between patients who underwent tracheostomy within 21 days (dotted line) and those who underwent tracheostomy later than 21 days (solid line; *P *< 0.001). **(b) **Difference in rates of successful weaning between patients with an arterial oxygen tension (PaO_2_)/fractional inspired oxygen (FiO_2_) ratio > 250 (dotted line) and those with a PaO_2_/FiO_2 _ratio < 250 (solid line; *P *< 0.001) before tracheostomy. **(c) **Difference in rates of successful weaning between the patients with post-tracheostomy pneumonia (solid line) and those without post-tracheostomy pneumonia (dotted line; *P *< 0.001)

In univariate analysis using the Kaplan–Meier method with log-rank test, reasons for intubation (pulmonary disease [*P *= 0.03] and lack of neurological disease [*P *< 0.01]), thrombocytopenia (*P *= 0.03), poor oxygenation before tracheostomy (*P *< 0.001), post-tracheostomy pneumonia during the weaning period (*P *< 0.001) and late tracheostomy (*P *< 0.001) were correlated with lower rates of successful weaning. A Cox regression model applied to the multivariate analysis showed that late tracheostomy, poor oxygenation and post-tracheostomy pneumonia during the weaning period were independent predictors of unsuccessful weaning (Fig. 3).

## Discussion

The present study demonstrated that patients who underwent tracheostomy and failed to wean from mechanical ventilation had longer intubation periods before tracheostomy. Timing of tracheostomy was correlated with length of ICU stay in the successful weaning group.

The type of ICU may also have an impact on the timing of tracheostomy. In surgical ICUs most patients do not have chronic lung disease or severe lung injury. These patients usually undergo tracheostomy early if they underwent a major surgical procedure and failed to extubate within several days after the operation. Previous studies [15-18] conducted in surgical ICUs have shown that tracheostomy performed within 1 week after intubation may be beneficial in lowering rates of pneumonia, and in shortening the duration of mechanical ventilation and length of ICU stay. However, other studies reported a higher incidence of ventilator-associated pneumonia [19,20] and longer length of ICU stay [21] in association with tracheostomy. In a neurological ICU, tracheostomy is usually performed if there is a depressed level of consciousness and poor ability to protect the airway. A recent study [22] demonstrated that early tracheostomy in patients in a medical ICU shortened the length of hospital stay and lowered hospital costs. The present study demonstrated that late tracheostomy may predispose to failure to wean and ICU mortality, especially when the intubation period is longer than 3 weeks. We also found that the duration of intubation before tracheostomy was correlated with length of ICU stay in patients who weaned successfully.

There were no obvious differences in terms of age, sex, APACHE II score, or underlying disease between the successful weaning and failure-to-wean groups, except for more neurological disease in the successful weaning group. However, in the 3 days before tracheostomy, higher WBC count, lower platelet count and lower PaO_2_/FiO_2 _ratio were noted in the failure-to-wean group. These observations suggest that leucocytosis, low platelet count and severity of respiratory failure before tracheostomy might have had a greater impact on outcome than initial presentation at ICU admission.

A longer intubation period was noted in those patients who failed to wean, indicating that, like the pre-tracheostomy conditions mentioned above, late tracheostomy may predispose to poor weaning outcome. A prolonged intubation period may impair the local barrier and bronchial hygiene, increasing the risk for bacterial colonization. Also, it may result in a higher rate of post-tracheostomy pneumonia – an association that was found in the failure-to-wean group. Ely and coworkers [23] demonstrated that prolonged intubation with mechanical ventilation was associated with increased hospital mortality and was independent of severity of illness. In the present study we found that prolonged intubation was associated with prolonged ICU stay. Delaying tracheostomy might not have been beneficial in these patients.

Reasons for intubation, poor pre-tracheostomy conditions, prolonged intubation and post-tracheostomy pneumonia were found to influence ventilator weaning in univariate analysis. However, in multivariate analysis we found that only late tracheostomy, pre-tracheostomy poor oxygenation and post-tracheostomy pneumonia during the weaning period were independent predictors of unsuccessful weaning. This finding suggests that timing of tracheostomy has an impact on ventilator weaning, as well as other clinical events. The 1989 ACCP Consensus Conference on Artificial Airways in Patients Receiving Mechanical Ventilation [10] suggested that tracheostomy is preferable if the anticipated need for mechanical ventilation is for more than 21 days. Recent ACCP guidelines [11] encourage early tracheostomy after patient stabilization if the patient needs prolonged mechanical ventilation. Our data support the suggestion of the earlier ACCP guidelines [10] that, when tracheostomy is performed more than 3 weeks after intubation, rates of ICU mortality and failure to wean increase.

The incidence of complications in adults who have undergone tracheostomy varies from 6% to 51% [4,24,25]. In the present study, the early complication rate was 38% and the late complication rate was 8% during hospitalization. The major early complication was minor to moderate bleeding from surgical wounds, which did not cause obvious clinical deterioration. We found tracheostomy to be a relatively safe procedure for airway management in patients who needed prolonged mechanical ventilation.

There are some limitations to the study. This retrospective study lacks baseline pulmonary function data before tracheostomy, which might have influenced the duration of weaning. Poor patient condition on admission to the medical ICU might have influenced the decision to perform a tracheostomy late.

## Conclusion

In this study we found that performance of tracheostomy more than 21 days after intubation was associated with prolonged weaning periods and low rates of successful weaning. It might also result in prolonged ICU stay. If one waits longer than 21 days, then it may be better to forego tracheostomy altogether.

## Key messages

• 	We found that performance of tracheostomy more than 21 days after intubation was associated with prolonged weaning periods and low rates of weaning.

• 	Late tracheostomy might also result in prolonged ICU stay; if one waits longer than 21 days, then it may be better to forego tracheostomy altogether.

## Abbreviations

ACCP = American College of Chest Physicians; APACHE = Acute Physiology and Chronic Health Evaluation; FiO_2 _= fractional inspired oxygen; ICU = intensive care unit; PaO_2 _= arterial oxygen tension; WBC = white blood cell.

## Competing interests

The author(s) declare that they have no competing interests.

## Authors' contributions

CLH participated in the study design and drafted the manuscript. KYC conceived the study, participated in its design and helped to draft the manuscript. JSJ, CJY and PCY participated in study design.

## Figures and Tables

**Table 1:** Demographic and clinical characteristics

Characteristics	Total (*n *= 163)	Successful weaning (*n *= 78)	Failure to wean (*n *= 85)	*P*
Age	70.3 ± 15.1	68.6 ± 15.4	71.8 ± 14.8	0.2
Sex (male/female)	93/70	49/29	44/41	0.2
APACHE II score	20.0 ± 7.2	19.6 ± 7.1	21.3 ± 7.6	0.1
Comorbid conditions				
Hypertension	63 (38.7%)	34 (43.6%)	29 (34.1%)	0.3
Diabetes mellitus	50 (30.7%)	27 (34.6%)	23 (27.1%)	0.3
COPD	34 (20.9%)	12 (15.4%)	22 (25.9%)	0.1
Malignancy	34 (20.9%)	14 (17.9%)	20 (23.5%)	0.4
Congestive heart failure	32 (19.6%)	19 (24.4%)	13 (15.3%)	0.2
Renal insufficiency	32 (19.6%)	15 (19.2%)	17 (20.0%)	1.0
Stroke	25 (15.3%)	16 (20.5%)	9 (10.6%)	0.09
Autoimmune disease	12 (7.4%)	5 (6.4%)	7 (8.2%)	0.8
Cancer metastatic to lung	8 (4.9%)	1 (0.01%)	7 (4.5%)	0.07

Shown are demographic data for 163 critically ill patients who underwent tracheostomy, and differences between patients who weaned successfully and those who failed to wean. APACHE, Acute Physiology and Chronic Health Evaluation; COPD, chronic obstructive pulmonary disease.

**Table 2:** Reasons for intubation

Reason for intubation	Total (*n *= 163)	Successful weaning (*n *= 78)	Failure to wean (*n *= 85)	*P*
Pulmonary disease	107	46 (59.0%)	61 (71.8%)	0.1
Infectious disease	18	7 (9.0%)	11 (12.9%)	0.5
Neurological disease	28	20 (25.6%)	8 (9.4%)	0.007
Circulatory disease	10	5 (6.4%)	5 (5.9%)	1.0

Shown are the reasons for intubation of the 163 patients who underwent tracheostomy, and differences between patients who weaned successfully and those who failed to wean.

**Table 3:** Pre-tracheostomy conditions

Parameter	Total (*n *= 163)	Successful weaning (*n *= 78)	Failure to wean (*n *= 85)	*P*
Albumin (g/dl)	2.7 ± 0.5	2.7 ± 0.4	2.6 ± 0.5	0.2
AST (U/l)	44.2 ± 49.6	47.5 ± 63.2	41.2 ± 32.5	0.4
Creatinine (mg/dl)	1.5 ± 1.7	1.4 ± 1.8	1.6 ± 1.6	0.6
Haemoglobin (g/dl)	10.5 ± 1.3	10.6 ± 1.3	10.4 ± 1.2	0.4
WBC count (cells/μl)	11993 ± 5474	11110 ± 4570	12803 ± 6104	0.05
Platelet (cell × 10^3^/μl)	192.2 ± 113.7	217.9 ± 116.3	168.7 ± 106.4	0.005
PaO_2_/FiO_2 _ratio	261.5 ± 93.6	284.5 ± 85.5	240.7 ± 96.1	0.003
pH	7.4 ± 0.1	7.4 ± 0.1	7.4 ± 0.1	0.2
Paco_2 _(mmHg)	40.9 ± 11.3	39.5 ± 7.5	42.2 ± 13.8	0.1

Shown are the pre-tracheostomy conditions in the 163 patients who underwent tracheostomy, and differences between patients who weaned successfully and those who failed to wean. AST, aspartate aminotransferase; CNS, central nervous system; FiO_2_, fractional inspired oxygen; PaO_2_, arterial oxygen tension; PaCO_2_, arterial carbon dioxide tension; WBC, white blood cell.

**Table 4:** Outcomes after tracheostomy

Outcomes	Total (*n *= 163)	Successful weaning (*n *= 78)	Failure to wean (*n *= 85)	*P*
ICU mortality (*n *[%])	31 (19.0%)	2 (2.6%)	29 (34.1%)	<0.001
In-hospital mortality (*n *[%])	78 (47.9%)	15 (19.2%)	63 (74.1%)	<0.001
Intubation period (days)	18.5 ± 10.9	16.3 ± 10.5	20.4 ± 10.9	0.02
Overall ICU stay (days)	29.7 ± 15.8	25.4 ± 13.8	33.7 ± 16.4	0.001
Post-tracheostomy ICU stay (days)	11.9 ± 9.9	9.7 ± 7.6	14.0 ± 11.3	0.005
Post-tracheostomy MV period (days)	27.3 ± 40.7	10.5 ± 12.6	42.6 ± 50.4	0.001
Post-tracheostomy pneumonia	109 (66.9%)	46 (59.0%)	63 (74.1%)	0.05

Shown are the outcomes after tracheostomy in the 163 patients who underwent tracheostomy, and differences between patients who weaned successfully and those who failed to wean. ICU, intensive care unit; MV, mechanical ventilation.
